# Cell Therapy for Chemically Induced Ovarian Failure in Mice

**DOI:** 10.1155/2014/720753

**Published:** 2014-12-04

**Authors:** Paula Terraciano, Tuane Garcez, Laura Ayres, Isabel Durli, Melchiani Baggio, Cristiana Palma Kuhl, Claudia Laurino, Eduardo Passos, Ana Helena Paz, Elizabeth Cirne-Lima

**Affiliations:** ^1^Hospital de Clínicas de Porto Alegre, 90035-903 Porto Alegre, RS, Brazil; ^2^PPGCV/UFRGS, 91540-000 Porto Alegre, RS, Brazil; ^3^PPGGO/UFRGS, 90035-007 Porto Alegre, RS, Brazil; ^4^PPGGASTRO/UFRGS, 90035-007 Porto Alegre, RS, Brazil

## Abstract

Cell therapy has been linked to an unexplained return of ovarian function and fertility in some cancer survivors. Studies modeling this in mice have shown that cells transplantation generates donor-derived oocytes in chemotherapy-treated recipients. This study was conducted to further clarify the impact of cell transplantation from different sources on female reproductive function after chemotherapy using a preclinical mouse model. *Methods*. Female mice were administered 7.5 mg/kg cisplatin followed by cell transplantation (one week later) using GFP+ female cell donors. For cell tracking, adipose derived stem cell GFP+ (ADSC), female germline stem cell GFP+/MVH+ (FGSC), or ovary cell suspension GFP+ mice were transplanted into cisplatin-treated wild-type recipients. After 7 or 14 days animals were killed and histological analysis, IHQ for GFP cells, and ELISA for estradiol were performed. *Results*. Histological examinations showed that ADSC, ovary cell suspension, and FGSC transplant increase the number of follicles with apparent normal structure in the cells recipient group euthanized on day 7. Cell tracking showed GFP+ samples 7 days after transplant. *Conclusion*. These data suggest that intraovarian injection of ADSCs and FGSC into mice with chemotherapy-induced ovarian failure diminished the damage caused by cisplatin.

## 1. Introduction

Premature ovarian failure is a syndrome characterized by lack of folliculogenesis and ovarian estrogen production, associated with amenorrhea and infertility in women under the age of 40 [1]. The syndrome is represented in 1% of menopausal women [2] and in 0.1% under the age of 30 [3]. One of the most devastating consequences of cancer treatment in the young female population is ovarian damage, resulting in diminished fertility potential [4]. Infertility and early menopause in young female adults have a high-level impact on patient self-esteem and quality of life. Adult stem cells have attracted considerable attention as tools for differentiation into several mesodermal lineages including osteoblasts, chondrocytes, adipocytes, cardiocytes, neural cells, and hematopoietic-supporting stroma and may therefore partly replace the impaired cells [5]. The clinical application of adipose-derived stem cells (ADSCs) as treatment for intractable diseases or traumatic tissue damage has attracted attention. Another kind of adult stem cells, named female germline stem cells (FGSCs), that produce oocytes* in vitro* and fertilization-competent eggs* in vivo* has been identified in and isolated from adult mouse ovaries and has been tested in several researches [6, 7]. Therefore, the aim of this study was to explore the therapeutic potency of intraovarian cell injection on ovarian function and structure in animal model of ovarian failure. We also investigated the restorative effects of ADSC, FGSC, or ovary cell suspension on ovarian function.

## 2. Methods

### 2.1. Animal Model

Healthy C57Bl/6 female mice were provided from FEPPS (Rio Grande do Sul Foundation of Production and Health Research) and used in the study. Animals between 7 and 8 weeks were used. C57Bl/6 wild-type were used as recipients and C57BL/6 GFP+ mice were used as donor cells. Ovarian failure was induced in the eight-week-old C57Bl/6 used as recipients by intraperitoneal injection of cisplatin (7.5 mg/kg in phosphate buffered saline (PBS)). Controls were injected with PBS. All procedures involving animals were approved by the Institutional Animal Care and Use Committee of Hospital de Clínicas de Porto Alegre and were conducted in accordance with the Guide for Care and Use of Laboratory Animals [8].

### 2.2. Preparation of Ovarian Tissue for Analysis

Ovaries were fixed in 10% buffered formalin and processed to prepare paraffin blocks, and serial sections of 5 mm were obtained and stained for H&E. Immunohistochemical staining for GFP cell tracking was performed.

### 2.3. Isolation and Characterization of Adipose Derived Stem Cells (ADSCs)

ADSCs were isolated from abdominal adipose tissue of C57Bl/6 GFP+ mice. Adipose tissue collected was degraded enzymatically in collagenase solution (1 mg/mL) and the cell suspension obtained was centrifuged and resuspended in DMEM with 1% antibiotic-antimycotic and 20% fetal bovine serum. Cells were plated and maintained in culture at 37°C with 5% CO_2_. The isolated cells developed to visible systematic colonies of adherent fibroblast-like cells at about 7–10 days after initial plating [9]. The ADSC differentiation potency was performed using specific protocols to induce differentiation into chondrocytes, adipocytes, and osteocytes [10] and flow cytometry assays were performed for CD29, CD11b, and CD34 markers.

### 2.4. Isolation of Female Germline Stem Cells (FGSCs)

Between 12 and 16 ovaries were collected for each experiment. FGSCs were isolated using the two-step enzymatic digestion method described previously [7, 11]. Briefly dissected ovaries were placed in PBS solution without calcium or magnesium and containing collagenase (1 mg/mL, type IV; Gibco, Aukland, NZ) and then incubated at 37°C with gentle agitation for 15 min. The ovarian tissue was then washed 2 to 4 times in PBS, placed in 0.05% trypsin EDTA, and incubated at 37°C for 10 min. When most of the cells were dispersed, trypsin was neutralized by adding cell culture medium supplemented with 10% fetal bovine serum (FBS). The suspension was centrifuged at 300 g for 5 min and the supernatant was carefully removed from the pellet. The pellet was resuspended and passed through a 70 *μ*m nylon cell strainer. These cells enriched with FGSCs were prepared by immunomagnetic isolation of MVH+ cells [6, 7]. For that goat anti-rabbit IgG microbeads (Miltenyi Biotec) were precoated with rabbit polyclonal MVH antibody (Abcam). MVH+ cells were selected by magnetic separation, according to the manufacturer's instructions.

### 2.5. Ovary Cell Suspension

Between 8 and 12 ovaries were collected for each experiment. Ovarian tissues were disaggregated using the two-step enzymatic digestion method described previously [7, 11]. The suspension was centrifuged at 300 g for 5 min and the supernatant was carefully removed from the pellet. The pellet was resuspended and cell clumps were avoided by passing the suspension through a 70 *μ*m nylon cell strainer.

### 2.6. Anesthetic and Surgical

**Figure 1:**
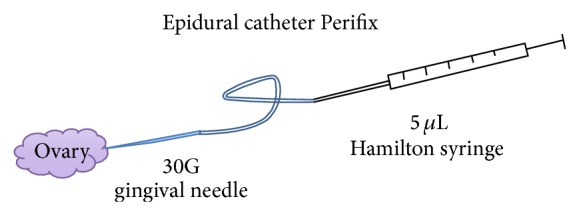
Schematic drawing: device for microinjection.

Anesthesia was induced and maintained with isoflurane vaporized oxygen administered by a mask. Analgesia was performed with premedication single dose of tramadol hydrochloride (10 mg*·*kg^−1^) intramuscularly (IM). Animals were placed in the left lateral position. Trichotomy and antisepsis (chlorhexidine gluconate 2%) were performed on the right flank from the last rib to the pelvic region. We proceeded to skin incision approximately 0.5 cm caudal to the last rib. The approach of the abdomen was performed by planes until the abdominal cavity. The right ovary was located and exposed using delicate surgical material. After exposure, the ovary was suspended for its capsule and a single cell suspension containing 1 × 10^4^ cell was injected (5 *μ*L). Cell injection was performed using a 30 G gingival needle attached to a microinjection device (see Figure 1) in a surgical microscope. Immediately after that we repositioned the ovary and proceeded to closing musculature. After that, procedure animals were kept under observation in an incubator with oxygen supply and heating until complete recovery from anesthesia.

### 2.7. Estradiol Levels

Blood sample was collected from retroorbital plexus during euthanasia procedure. Serum was separated by centrifugation and stored at −80°C. Estradiol (E2) serum levels were measured by enzyme-linked immunosorbent assay (ELISA) (Uscn Life Science Inc., Houston, Texas, EUA).

### 2.8. Experimental Design

48 female mice C57Bl/6 wild-type were divided into 8 groups (*n* = 6) and euthanasia was performed in 7 or 14 days after cell transplantation: ADSC 7 or 14 days (A7, A14), FGSC 7 or 14 days (F7, F14), ovary suspension 7 or 14 days (O7, O14), and control 7 or 14 days (C7, C14). One week after ovarian failure induced by intraperitoneal injection of cisplatin (7.5 mg/kg) animals were intraovarian injected with 5 *μ*L volume containing 1 × 10^4^ ADSC, FGSC, ovary cell suspension, or PBS (control group).

### 2.9. Statistical Analysis

We performed logistic regression to derive estimates of the probability of viable follicles in these sections of the ovaries.

## 3. Results

### 3.1. Animal Model

To verify the follicle depletion we performed histological analysis of the ovaries. H&E-stained sections of ovaries showed a lower number of viable follicles in chemotherapy-treated ovaries (Figure 4(a))

### 3.2. Isolation and Characterization of Cells

**Figure 2:**
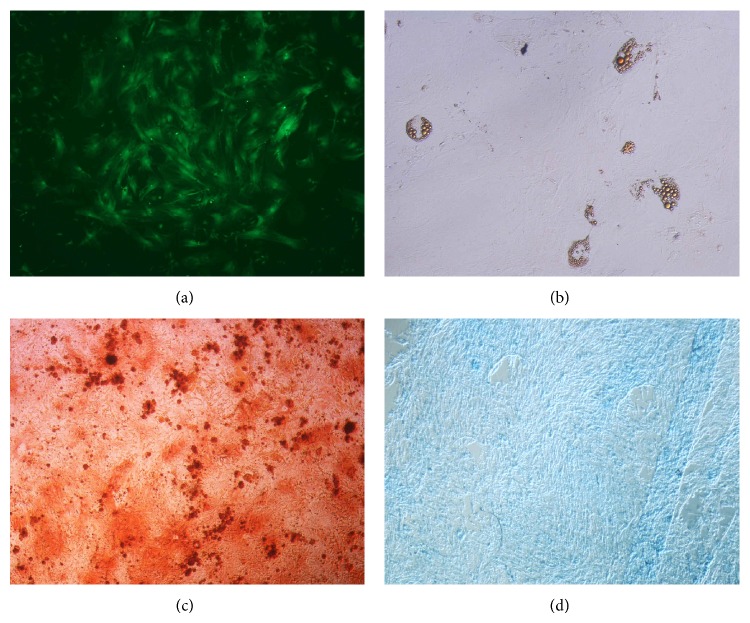
(a) Epifluorescence microscopy photomicrograph ADSC GFP+ positive cells culture 40x. (b) Adipocyte differentiation Oil Red staining 20x. (c) Osteocyte differentiation Alizarin Red staining 40x. (d) Chondrocyte differentiation Alcian Blue staining 40x.

**Figure 3:**
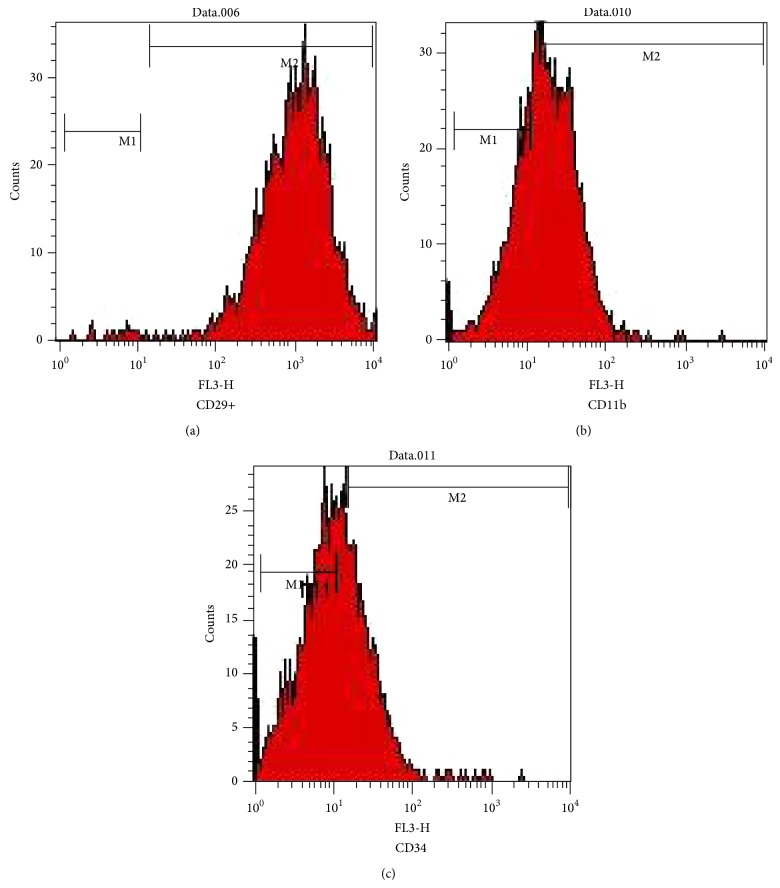
Flow cytometry assay, cells stained with MSCs cell surface antigen markers: (a) CD29+ (b), CD11b−, and (c) CD34−.

The ADSC cell population in culture was characterized by its adhesiveness and fibroblastoid shape and showed ability to* in vitro *differentiate into adipocytes, osteocytes, and chondrocytes (Figure 2). Cells were evaluated for expression of CD29, CD34, and CB11b by flow cytometer. The majority of cells were negative for CD34 and CD11b and positive for CD29 (Figure 3).

### 3.3. Histological Analysis

**Figure 4:**
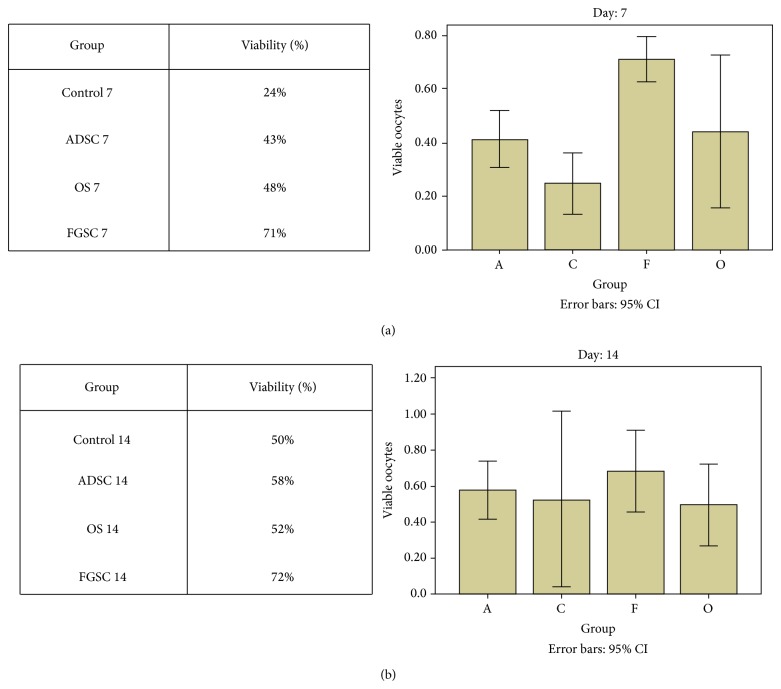
(a) Probability of viable cells in groups euthanized on day 7. (b) Probability of viable cells in groups euthanized on day 14.

**Figure 5:**
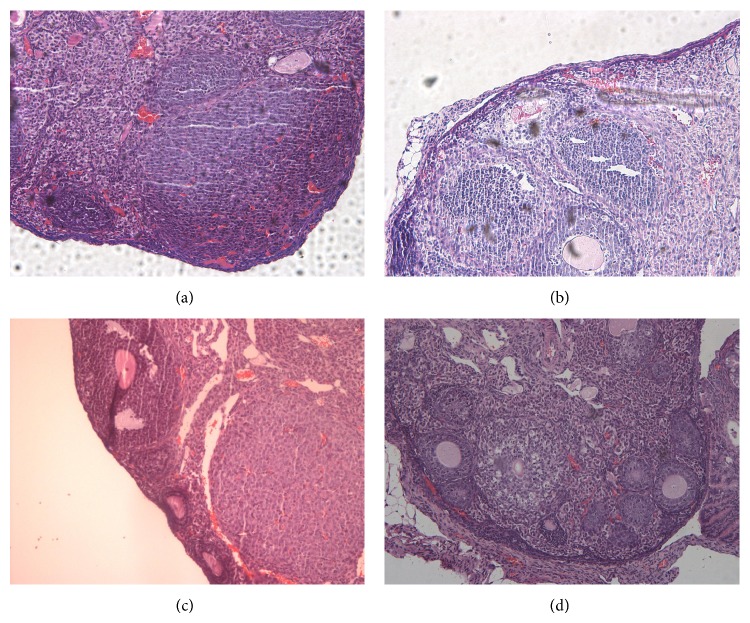
Photomicrograph H&E stained from histological ovaries sections: experimental groups euthanized on day 7. (a) Control 40x, (b) ovary suspension transplant 100x, (c) ADSC transplant 100x, and (d) FGSC transplant 40x.

**Figure 6:**
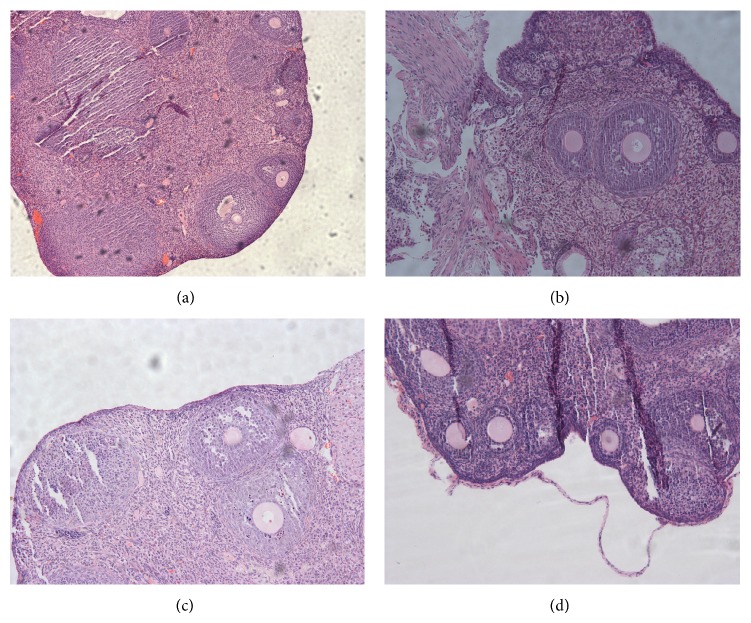
Photomicrograph H&E stained from histological ovaries sections: experimental groups euthanized on day 14. (a) Control 40x, (b) ovary suspension transplant 200x, (c) ADSC transplant 200x, and (d) FGSC transplant 100x.

ADSC, FGSC, and ovary cell suspension groups euthanized on day 7 after transplantation had a significant improvement compared to control. There was higher probability of follicles viable cells (71%) in FGSC group followed by OS (48%) and ADSC (43%) (Figures 4 and 5). We also observed a tendency to better recovery in group FGSC compared to the other groups 14 days after transplantation, although this difference was not statistically significant (Figures 4 and 6). There was no difference in estradiol serum levels between all groups (data not shown).

## 4. Discussion

Stem cell-based strategies for ovarian regeneration and oocyte production have been proposed as future clinical therapies for treating infertility in women [7, 12]. MSCs have attracted interest for their possible use for both cell and gene therapies because of their capacity for self-renewal and multipotentiality for differentiation [13].

Our results demonstrate that transplantation of stem cells promotes better recovery in terms of viable oocytes in groups receiving ADSC and OS compared to the control group. We believe that both cell types, ADSC and OS, have similar results based on the theory postulated by da Silva Meirelles et al. in 2006 [14] claiming that the mesenchymal stem cells reside in all postnatal organs and tissues in a perivascular niche. Therefore we believe that ovary suspension cells have the same antiapoptotic, immunomodulatory, chemoattraction, and angiogenic characteristics that ADSC possess related its perivascular location described by Meirelles et al. [15].

One of the mechanisms behind this is the paracrine mediators secreted by MSC which might be involved in the repair by preventing cell apoptosis and promoting functional recovery [16]. However, currently little information is available regarding the therapeutic potential of MSC for chemotherapy-induced ovarian damage. In this study we have demonstrated that ADSC intraovarian transplantation improves ovarian function, suggesting that this procedure may be useful in patients with ovarian failure. These results are in agreement with Abbasy et al. (2010) [17] and Abd-Allah et al. (2013) [12] because they suggest that MSC implantation can ameliorate ovarian function in hormonal and follicular development abilities in rats with ovarian failure.

Fu et al. 2008 [18], using bone marrow derived stem cells, demonstrated that MSC secretes cytokines* in vitro*, including VEGF, IGF-1, and HGF, and inhibits chemotherapy-induced apoptosis of GC* in vitro* by upregulating Bcl-2 protein and cytokines and, in a rat model of chemotherapy-induced ovarian damage, MSC transplantation reduces cell apoptosis and improves ovarian function.

**Figure 7:**
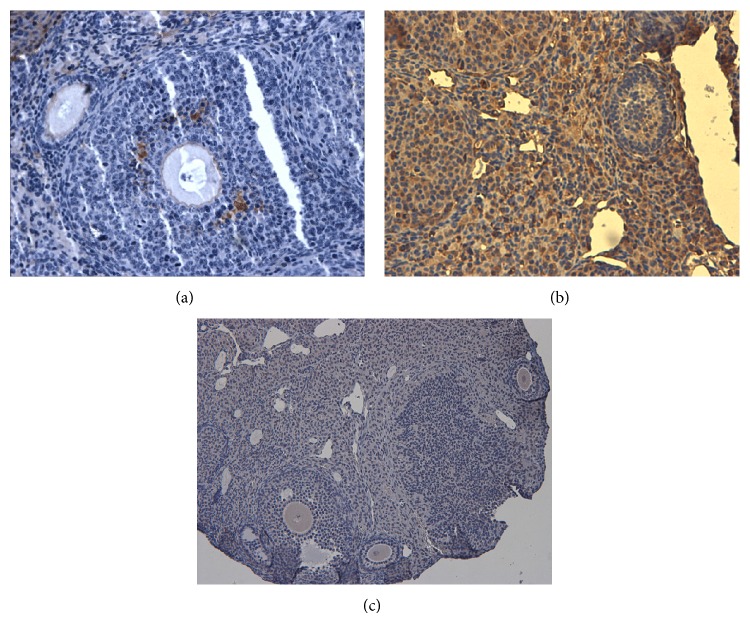
IHQ for GFP cell tracking. (a) GFP cells in ovary 7 days after transplant. (b) Negative control. (c) Positive control.

Takehara et al. 2013 [19] investigated the restorative effects on ovarian function and the safety of adipose-derived stem cells (ADSCs). ADSCs were shown to be capable of inducing angiogenesis and restoring the number of ovarian follicles and corpus lutea in ovaries. In addition, the localization of the Y chromosome was investigated using the fluorescent in situ hybridization method by injecting male ADSC into the ovaries; as a result, the Y chromosomes were localized not in the follicles but in the thecal layers. In the same way as Takehara et al., 2013 immunohistochemistry assay for GFP cell tracking, in our study, found the GFP positive cells distributed in thecal layer area (Figure 7(a)), which indicates that ADSC transplanted into the ovary may not differentiate directly into oocytes or granulosa cells but may survive in the interstitium, playing important accessory roles in the microenvironment surrounding the oocytes in the ovary.

These findings suggest that MSCs may have a role in restoring damaged ovarian function and could be useful for regenerative medicine. Although ADSC transplantation has shown an improvement in viable follicles, in this study, FGSC transplantation showed better results on days 7 and 14 of euthanasia. Despite the concept of the finite and nonrenewable stock of germ cells which is considered a basic premise of reproductive physiology for over 150 years [19], Johnson et al. 2004 [6, 20] presented evidence to propose a revision of this paradigm, showing indications of oogenesis and folliculogenesis in the postnatal period, due to stem cells characteristics, like the ability to generate cells of other tissues.

This potential stem cell plasticity appears to favor the hypothesis of neo-oogenesis/folliculogenesis formulated by Johnson et al. [6]. These authors demonstrated the presence of specific proteins of meiosis in ovaries of adult mice, which would occur only during the fetal stage, according to the current concept. In the same study, the authors mention the reduction of atretic follicles as indicative of a significant hemodynamic activity in the female gonad, suggesting a more dynamic mobile traffic in the ovaries. The contrast of these findings and conclusions to previous concepts in the field resulted in considerable skepticism by some members of the scientific and medical communities [2, 21–23]. Nonetheless, a rapidly growing number of studies have subsequently confirmed that ovaries of adult mice contain a rare population of mitotically active germ cells that can be isolated and propagated in culture for months, and that gives rise to oocytes* in vitro* and upon transplantation into ovaries of recipient mice* in vivo* [24–26]. In our study we also isolate germ cells, by immunomagnetic separation, and transplant them to the ovary. Our results are consistent with several researches that use these stem cell types and showed better results in folliculogenesis [26, 27]. Although our analysis has focused on the evaluation of follicular viability, we can speculate that the group treated with MVH cells showed better results in comparison to groups ADSC and OS because the ability of folliculogenesis MVH was demonstrated in the studies mentioned above. As opposed to Abd-Allah et al. 2013 [12] we found no statistical difference in serum estradiol levels. We attribute this discrepancy to the use of different animal models and different analysis times.

In addition to opening a new research field in human reproductive biology, which was inconceivable less than ten years ago, clear evidence for the existence of these cells in women may offer new opportunities to enhance current fertility preservation strategies. For example, with assisted reproductive technologies involving cryopreservation of ovarian cortical tissue already in development for female cancer patients [28, 29] isolation and expansion of FGSC from this tissue, before or after cryopreservation, might be useful for new fertility applications. In addition, the availability of a detailed protocol for purification of these newly discovered cells from human ovary tissue provides us with a much more physiologically relevant* in vitro* model system to study human female germ cell development instead of embryonic stem cell which is currently used as models for human female gametogenesis [30, 31].

## 5. Conclusions

Our findings in this study suggest potential therapeutic effects for ovarian dysfunction by intraovarian injection of ADCs and FGSC. And the transplant of those cells may have a role in restoring damaged ovarian tissue induced by cisplatin.

Furthermore, the results obtained specifically with FGSC therapy suggested that the ovarian stem cells should be a candidate for future ovarian failure restoration because it provides a better clinical outcome, probably considering it a committed stem cell with the ovarian microenvironment offering it a higher capability to specifically enhance ovarian tissue repair.
